# Assessments of Drought Impacts on Vegetation in China with the Optimal Time Scales of the Climatic Drought Index

**DOI:** 10.3390/ijerph120707615

**Published:** 2015-07-08

**Authors:** Zheng Li, Tao Zhou, Xiang Zhao, Kaicheng Huang, Shan Gao, Hao Wu, Hui Luo

**Affiliations:** 1 State Key Laboratory of Earth Surface Processes and Resource Ecology, Beijing Normal University, Beijing 100875, China; E-Mails: zhengli@mail.bnu.edu.cn (Z.L.); huangkaicheng@mail.bnu.edu.cn (K.H.); gsapril@163.com (S.G.); wuhaogis@163.com (H.W.); luohui3377@163.com (H.L.); 2 Academy of Disaster Reduction and Emergency Management, Ministry of Civil Affairs & Ministry of Education, Beijing 100875, China; 3 School of Geography, Beijing Normal University, Beijing 100875, China; E-Mail: zhaoxiang@bnu.edu.cn

**Keywords:** drought, SPEI, optimal time scales, NDVI, ecological assessment, China

## Abstract

Drought is expected to increase in frequency and severity due to global warming, and its impacts on vegetation are typically extensively evaluated with climatic drought indices, such as multi-scalar Standardized Precipitation Evapotranspiration Index (SPEI). We analyzed the covariation between the SPEIs of various time scales and the anomalies of the normalized difference vegetation index (NDVI), from which the vegetation type-related optimal time scales were retrieved. The results indicated that the optimal time scales of needle-leaved forest, broadleaf forest and shrubland were between 10 and 12 months, which were considerably longer than the grassland, meadow and cultivated vegetation ones (2 to 4 months). When the optimal vegetation type-related time scales were used, the SPEI could better reflect the vegetation’s responses to water conditions, with the correlation coefficients between SPEIs and NDVI anomalies increased by 5.88% to 28.4%. We investigated the spatio-temporal characteristics of drought and quantified the different responses of vegetation growth to drought during the growing season (April–October). The results revealed that the frequency of drought has increased in the 21st century with the drying trend occurring in most of China. These results are useful for ecological assessments and adapting management steps to mitigate the impact of drought on vegetation. They are helpful to employ water resources more efficiently and reduce potential damage to human health caused by water shortages.

## 1. Introduction

Drought is a complex natural hazard that affects water resources, natural ecosystems, agriculture, and society more frequently than any other natural disaster [1,2,3]. Over 900 million people worldwide were affected by drought during the period of 1999 to 2010 [4]. Drought has occurred in most places of the world, including wet and humid regions [5,6], and it is expected to increase in frequency and severity due to decreasing precipitation and increasing evapotranspiration driven by global warming [7,8,9,10]. In China, drought has become one of the most severe natural disasters [6], and the affected area has increased in the past 50 years [11]. Serious drought events occur frequently in China, especially in Yunnan Province [12,13]. Many efforts have been made to develop methodologies to assess the drought. The most widely used method is to propose a climatic drought index, which is simple to use to reflect the fluctuation of water conditions [14,15,16].

There are several climatic drought indices that are widely used for evaluating drought conditions, including the Palmer Drought Severity Index (PDSI) [17], the Standardized Precipitation Index (SPI) [18] and the Standardized Precipitation Evapotranspiration Index (SPEI) [19]. The PDSI is based on the supply and demand concept of the water balance equation; however, the primary limitation of the PDSI is that it has a fixed temporal scale and autoregressive characteristics [20]. The SPI is a probabilistic precipitation approach that is based on the cumulative precipitation available and incorporates different time scales [21], but it only involves precipitation and does not consider other variables (e.g., temperature and evapotranspiration), which can potentially affect the frequency of drought [19]. The SPEI, however, is based on the monthly water balance derived from both precipitation and potential evapotranspiration (PET), and it can be compared with the self-calibrated PDSI [22].

The main advantages of SPEI over other climatic drought indicators are its ability to identify the evapotranspiration and temperature variability in the drought assessments [23]. The SPEI is more appropriate to investigate the characteristics of drought in that it is temporally flexible and spatially consistent, and reflects the water deficits at different time scales; thus, it has become a significant tool to assess moisture conditions [24]. However, due to the complexity of different vegetation’s responses to water balance at different time scales, the SPEI at fixed time scales (e.g., 12-month time scale, SPEI-12) is usually applied to investigate the drought trend [15,25], which tends to produce system biases when the region contains different types of vegetation.

As the impact of drought on the growth of vegetation varies among different species and leads to distinct responses according to the different time scales considered [26,27], it is essential to determine the vegetation type-specific optimal time scale of SPEI for each type of vegetation before assessing drought by SPEI. Additionally, although the climatic drought indicators are important tools to investigate the extent of drought, they contain no vegetation-specific information that might directly reflect different physiological strategies for coping with water deficits. Thus, it is necessary to build a linkage between the climatic drought index and the vegetation’s response index. An appropriate SPEI with an optimal time scale should have a good correlation between the SPEI and physiological drought-related indicators. The normalized difference vegetation index (NDVI) is widely used to monitor the vegetation activity and its anomaly is a good surrogate to reflect vegetation’s physiologic drought conditions [28,29,30]. As a result, through the comparison of correlation coefficients between the SPEIs of various time scales and the anomaly of NDVIs, the vegetation type-related optimal time scales could be revealed. That is, the SPEI with the optimal time scale should have the strongest correlation with the NDVI anomalies.

In this study, we aimed to estimate the vegetation type-related optimal time scales of SPEI by analyzing the covariation between the SPEIs of various time scales and NDVI anomalies. After that, based on these estimated optimal time scales of SPEI (SPEI_opt_), the spatio-temporal characteristics of drought were investigated and then compared with those derived from the SPEI-12. Finally, regression models were built to compare the responses of vegetation to the drought based on both the SPEI-12 and the SPEI_opt_ during the growing season (April to October).

## 2. Data and Methods

### 2.1. Climatic Data and Multi-Scalar SPEI Drought Indicator

The climatic data employed in this study include the monthly average temperature (MAT) and the monthly precipitation (P) of 505 meteorological stations across the entirety of China from 1960 to 2013. These precipitation and temperature data are collected from the National Climate Center of the Chinese Meteorological Administration (CMA). The homogeneity and reliability of the monthly meteorological data are checked and firmly controlled by the CMA before its release [15]. This study applies 5-year running means before and after the missing data to ensure that the time series are continuous. The stations with missing meteorological data for more than 12 consecutive months are rejected and not used in this study.

**Figure 1:**
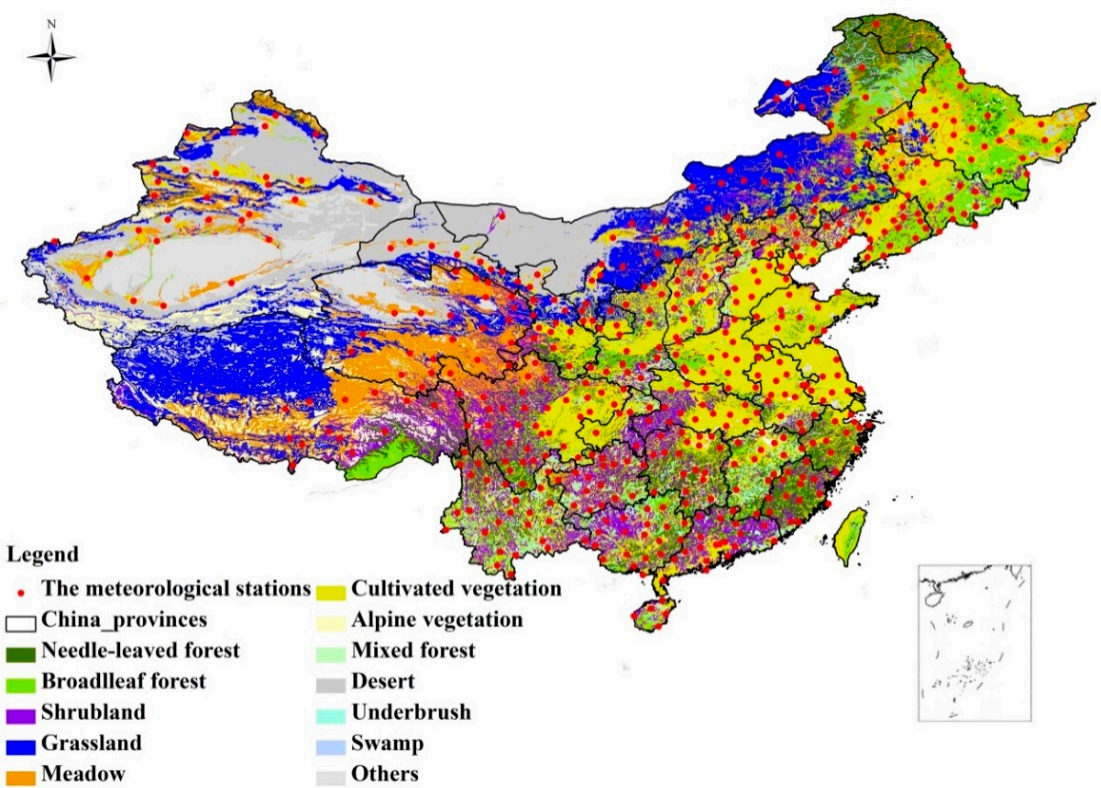
The distribution of the meteorological stations and the major vegetation types of China.

The SPEI is a site-specific drought indicator with multi-time scales. The calculation of SPEI is based on the monthly average temperature and precipitation. There are three major steps to calculate the SPEI with different time scales [19,31]. The first is to calculate the potential evapotranspiration (PET) based on the scheme of Thornthwaite [32]. The second is to calculate the water balance at various time scales (P-PET). The last is to normalize the water balance into a log-logistic probability distribution to obtain the SPEI series. The SPEI time series obtained for different time scales represent the cumulative water balance over the previous *n* months [33,34], and this study considers the time scales from 1 to 12 months to cover the impact of the inter-annual variability of SPEI on vegetation. As the SPEI is a standardized value, it could be compared with other SPEI values over time and space, and also allows one to determine the patterns of spatial and temporal variability, trend and extent of drought [23,35]. The drought categories (*i.e.*, groups) based on SPEI values are listed in Table 1 [15,24,34]. The time scales of SPEI are separated into three groups: short time scales (1–4 months), medium time scales (5–9 months) and long time scales (10–12 months) [36]. The study period is from 1982 to 2011 to match the time range of NDVI dataset. The representative vegetation type for each meteorological station is determined from a 1:1,000,000 vegetation map of the People’s Republic of China [37], and the 505 meteorological stations cover most regions in China with six major land cover types, including needle-leaved forest, broadleaf forest, shrubland, grassland, meadow and cultivated vegetation (Figure 1).

**Table 1:** The drought categories based on SPEIs.

SPEI	Drought Category	Probability
≥2.00	Extreme wet	0.02
[1.50, 2.00)	Severe wet	0.06
[1.00, 1.50)	Moderate wet	0.10
(−1.00, 1.00)	Normal	0.65
(−1.50, −1.00]	Moderate drought	0.10
(−2.00, −1.50]	Severe drought	0.05
≤−2.00	Extreme drought	0.02

### 2.2. Remote Sensing Data

The normalized difference vegetation index (NDVI) derived from remote sensing data is defined as the ratio of the difference between near-infrared reflectance and red visible reflectance to their sum [38]. It is widely used to detect the spatio-temporal characteristics of drought [26,39,40,41,42,43,44]. The Global Inventory Monitoring and Modeling Systems (GIMMS3g) NDVI dataset used in this study is produced from the National Oceanographic and Atmospheric Administration (NOAA) with the Advanced Very High Resolution Radiometer (AVHRR) instruments [45]. During the pre-processing, data with large solar zenith angle are excluded and maximum value composite algorithm is used to obtain NDVI for each two week period [46]. The calibration is employed to alleviate known limitations of the AVHRR measurements induced by intersensor calibration, orbital drift and atmospheric contamination [38,47]. The GIMMS3g NDVI dataset has a spatial resolution of 8 km and a temporal resolution of 15 days [48], and it has been widely used for global vegetation monitoring due to its longest time range [49,50]. The quality and consistency of this dataset are assured by correcting for sensor degradation, sensor inter-calibration differences, solar zenith and viewing angles, volcanic aerosols, atmospheric water vapor and cloud cover [33]. To correspond with the temporal resolution of site-specific SPEI time series, the NDVI series are transformed into a monthly composited dataset based on the maximum value composite (MVC) method. The remote sensing data (NDVI) is extracted by the point that was corresponding to the coordinates of the meteorological stations. The extracted value of a specific meteorological station in the remote sensing data is the central value of the pixel that the point is located. As we directly analyze the relationship between the station-based SPEI and the corresponding NDVI, it could avoid some potential uncertainties caused by spatial interpolation of climate observations.

As the variation of NDVI is widely used to monitor drought-related environmental stress, we develop an NDVI anomaly index, NDVI_anomaly_, to reflect the variation of vegetation activity. For a specific meteorological station:

(1) $${\text{NDVI}}_{\text{anomaly}}\left(y,m\right)=\frac{NDVI_{value}\left(y,m\right)-NDVI_{avg}\left(m\right)}{\sqrt{\frac{1}{N}\sum_{n=1}^{N}{\left(NDVI_{value}\left(y,m\right)-NDVI_{avg}\left(m\right)\right)}^{2}}}$$

where y and m separated represent the year and the month, $NDVI_{value}\left(y,m\right)$ represents the value of NDVI for a specific year and month, $NDVI_{avg}\left(m\right)$ represents the average value of the NDVI for a special month, *N* is the total year from 1982 to 2011 (*N* = 30). The calculation of NDVI_anomaly_ is based on the ratio of the differences between the NDVI value and the corresponding NDVI averaged value (the multi-year mean of the specific month) to the standard deviation. As a result, the mean of NDVI_anomaly_ is 0 and the standard deviation of NDVI_anomaly_ is 1. As the NDVI_anomaly_ value is the standardized value, it could be compared in both time and space.

### 2.3. Drought Assessment Index

There are two widely used drought assessment indices; one is the ratio of meteorological stations involved droughts (*P*_i_), and the other is the drying trends based on each meteorological station (*a_j_*). These indices can better reflect patterns of spatial and temporal variability, trend and frequency of drought on a large scale [51].

The ratio of meteorological stations involved droughts (*P_i_*) is employed to assess the frequency of drought and the extent of drought impact on vegetation growth over temporal evaluation:

(2) $$P_{i}=\frac{m}{M}\;\times \;100\%$$

where *m* is the number of meteorological stations that involved droughts based on SPEI (SPEI ≤ −1) [34,52], *M* is the number of all the meteorological stations (*M* = 505), and *i* is the specific year during 1982 to 2011.

The meteorological station-based drying trend (*a_j_*) is used to reflect the drying trends across China over spatial distribution:

(3) $$\text{y}\left(\text{j}\right)=a_{j}\times \text{ x}\left(\text{j}\right)+b_{j}$$

where *j* is the meteorological station, x is the time series of SPEI of each meteorological station, and *a_j_* and *b_j_* are the regression coefficients.

### 2.4. Statistical Methods

As the calculated value of SPEI depended on the time scales of water balance, it was necessary to consider the rationality of the selected time scale on the drought responses of various vegetation types. To determine the vegetation type-specific time scale, that is, the optimal time scales of various vegetation types, we should combine the climatic drought indicator (e.g., SPEI) determined by climatic factors with the physiological drought indicator of vegetation (e.g., NDVI anomaly) determined by water stress.

Based on the multi-scalar SPEI (1–12 months) and the monthly NDVI_anomaly_ (April–October) during the growing season at different meteorological stations, the Pearson correlations were conducted for 7 monthly NDVI anomaly series (*i.e.*, April–October) *vs.* 12 SPEI series (*i.e.*, SPEIs with the time scale of 1-, 2-, …, and 12-month). That is, there were 84 correlation coefficients for each meteorological station. The correlation coefficients that passed the significance test (*p* < 0.05) and their corresponding time scales were kept for further study. The meteorological stations were grouped by the vegetation types. For each vegetation type, the highest frequency of the time scale of SPEI with the maximum correlation coefficients for each meteorological station was picked up as the optimal time scale (SPEI_opt_) for this vegetation type.

The optimal time scales of various vegetation types (SPEI_opt_) were compared with the fixed 12 month time scale (SPEI-12) to show the degree of the responses of vegetation to the drought. To analyze the spatio-temporal patterns of drought at annual scale, the NDVI value which could represent the optimum growth conditions of vegetation activity in a year should be employed to show the vegetation activity. The maximum NDVI value a year mainly concentrated in growing season, and could better reflect the exuberant vegetation activity [53]. The drought assessment indices based on the SPEI time series were used to assess the characteristics of the drought. The regression models for different vegetation types were compared based on the relationship between the anomaly of NDVI and the SPEI with both the fixed (SPEI-12) and the vegetation type-dependent optimal time scale (SPEI_o__pt_).

## 3. Results

### 3.1. Drought Derived from the SPEI-12

**Figure 2:**
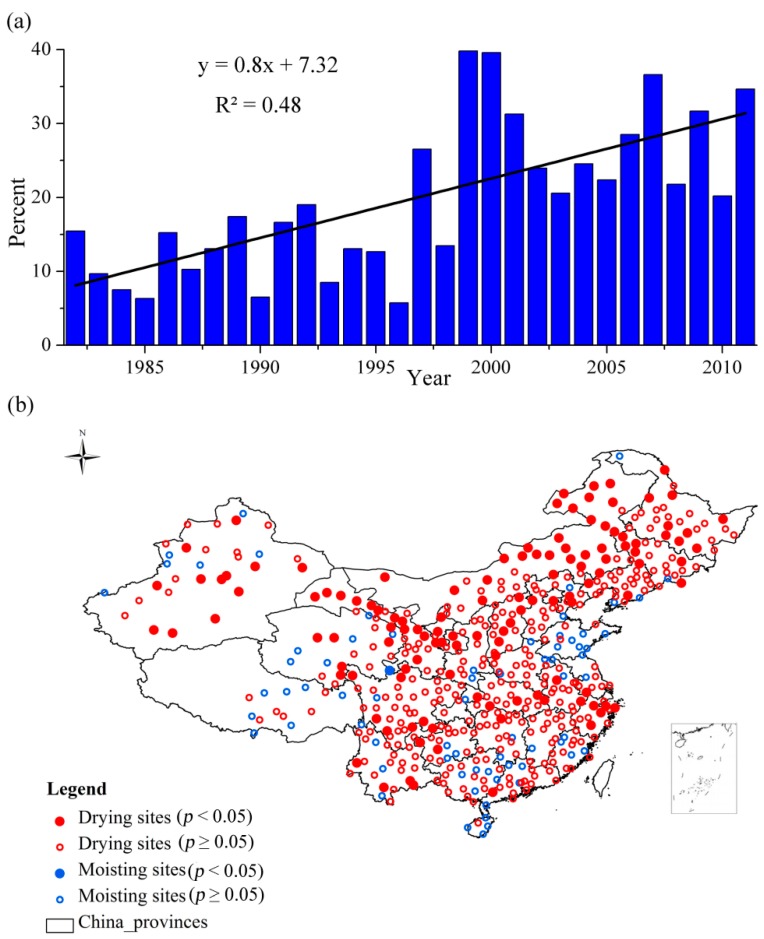
(**a**) The frequency of drought from 1982 to 2011 based on the SPEI-12; (**b**) The drying trend based on the SPEI-12 time series for each meteorological station over China.

The SPEI with a specific time scale was helpful to monitor the drought in that it considered the cumulative water balance. However, many studies [25,54] used a fixed 12 month time scale of SPEI (SPEI-12) as it is simply calculated to evaluate the drought. Figure 2a showed the frequency of drought from 1982 to 2011 based on the ratio of the number of meteorological stations when the SPEI was less than −1 (*i.e.*, SPEI ≤ −1) to the whole number of the meteorological stations.

The value of −1 was a critical value of drought (Table 1). When the SPEI of a specific meteorological station was less than −1, it was regarded as the drought. The temporal evaluation of drought based on the *P_i_* showed the frequency of drought has increased especially from the beginning of the 21st century (Figure 2a). Figure 2 showed the drying trend based on the SPEI time series (SPEI-12) over China for each meteorological station. For a specific meteorological station, if the SPEI time series had a decreased trend, it meant that it had a drying trend, but the value of SPEI was not necessarily less than −1 (for example, from severe wet to moderate wet). The spatial evaluation of drought based on the *a_j_* showed that the drying trend and extent have covered most of China (Figure 2b). Approximately 434 out of 505 meteorological stations, distributed in most regions in China, exhibited a drying trend. The meteorological stations that passed the significance test (*p* < 0.05) were concentrated in Northern China. However, Southern China also exhibited the drying trend, although most of the stations did not pass the significance test. The regions that had wetting trends were mainly located at regions in the east part of central China and Southeast China.

### 3.2. Drought Derived from the Optimal Time Scales of SPEI

The SPEI with the optimal time scales (*i.e.*, SPEI_opt_) considered the characteristics of different vegetation types and their sensitivity to accumulated water balance. The statistics of the optimal time scales of SPEI and the related season for various vegetation types are shown in Table 2. The optimal time scales of the needle-leaved forest, the broadleaf forest and the shrubland were separately 11, 10, 12 months, and they all were related to the long time scales (10–12 months). For the grassland, the meadow and the cultivated vegetation, the optimal time scales were 3, 4, and 2 months, respectively, and they responded to the short time scales (2–4 months).

**Table 2:** The optimal time scales of SPEIs for different vegetation types.

Vegetation Types	Number of Stations	Optimal Time Scales (months)	Season
Needle-leaved forest	39	11	Summer
Broadleaf forest	26	10	Summer
Shrubland	46	12	Summer
Grassland	39	3	Summer
Meadow	32	4	Autumn
Cultivated vegetation	323	2	Summer

As differences exist in the SPEI time scales for various vegetation types, the correlation coefficients between the SPEIs and the NDVI_anomaly_ also exhibited discrepancies. The correlation coefficients based on the corresponding optimal SPEI time scales (SPEI_opt_) for the vegetation types increased compared with the fixed SPEI time scale (SPEI-12) (Table 3).

**Table 3:** The improvements of correlation coefficients for SPEI_opt_.

Vegetation Types	Increased Proportion of the Correlation Coefficients (%)
Needle-leaved forest	5.9
Broadleaf forest	10.6
Shrubland	0
Grassland	20
Meadow	16.3
Cultivated vegetation	28.4

All the vegetation types except the shrubland exhibited increased correlation coefficients. As the optimal time scale of shrubland was 12 months, there was no difference in the correlation coefficients for shrubland. For the forests, the increased proportion of the correlation coefficients were separately approximately 5.9% and 10.6% compared with the SPEI-12. The forests had similar optimal time scales (the needle-leaved forest: 11 months; the broadleaf forest: 10 months) compared with the SPEI-12, and the differences of SPEI values were small among the long time scales (10–12 months). For the grassland, the meadow and the cultivated vegetation, the optimal time scales were 3 months, 4 months and 2 months, respectively. The optimal time scales for these three vegetation types were much shorter than the SPEI-12, and the increased proportion of the correlation coefficients were 20%, 16.3% and 28.4%. In sum, the SPEI with optimal time scale (SPEI_opt_) had better responses to drought than the fixed 12-month SPEI (SPEI-12), especially for the grassland, meadow and cultivated vegetation.

**Figure 3:**
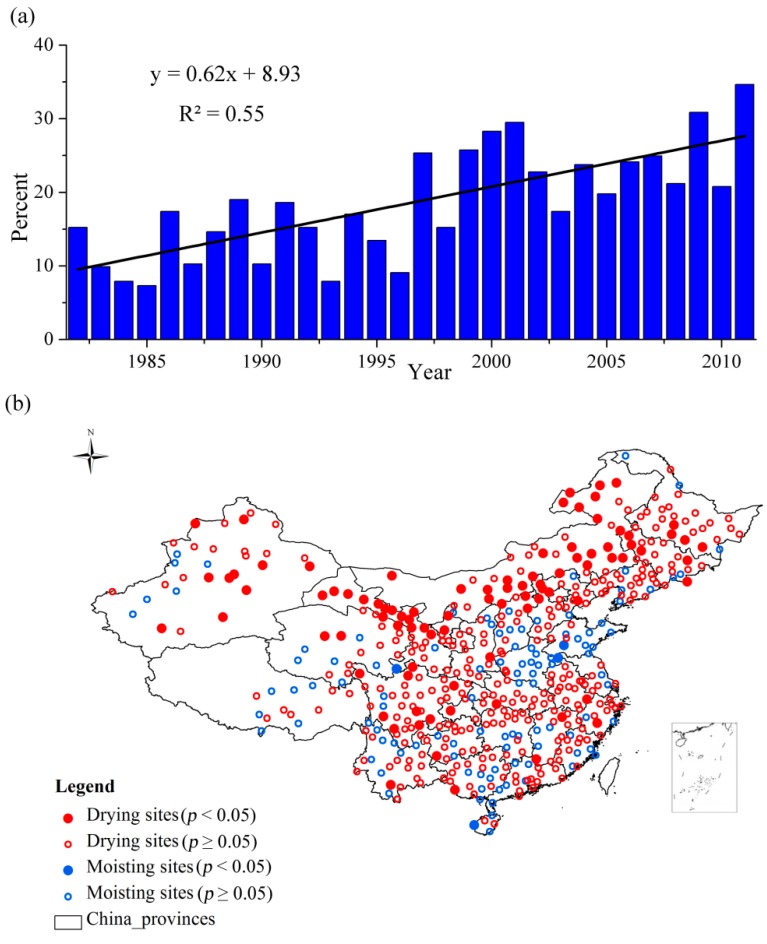
(**a**) The frequency of drought from 1982 to 2011 based on the SPEI_opt_; (**b**) The drying trend based on the time series of SPEI_opt_.

The vegetation type-dependent SPEI_opt_ could provide us with more specific and comprehensive understanding of the spatio-temporal characteristics of drought (Figure 3). From the perspective of temporal evolution, the frequency of drought has increased especially from the 21st century during the period 1982–2011 (Figure 3a).

The R^2^ based on the SPEI_opt_ was approximately 7% higher in R^2^ than that derived from the SPEI-12. From the perspective of spatial distribution of drought in China, the drying trend was obvious during the period from 1982 to 2011 (Figure 3b). The 395 out of 505 meteorological stations exhibited the drying trend. The meteorological stations that passed the significance test (*p* < 0.05) were mostly concentrated in Northern China compared with the SPEI-12. Southern China also had a drying trend, which was not as widely spread as that based on the SPEI-12.

### 3.3. Comparison of Large-Scale Drought Events

**Figure 4:**
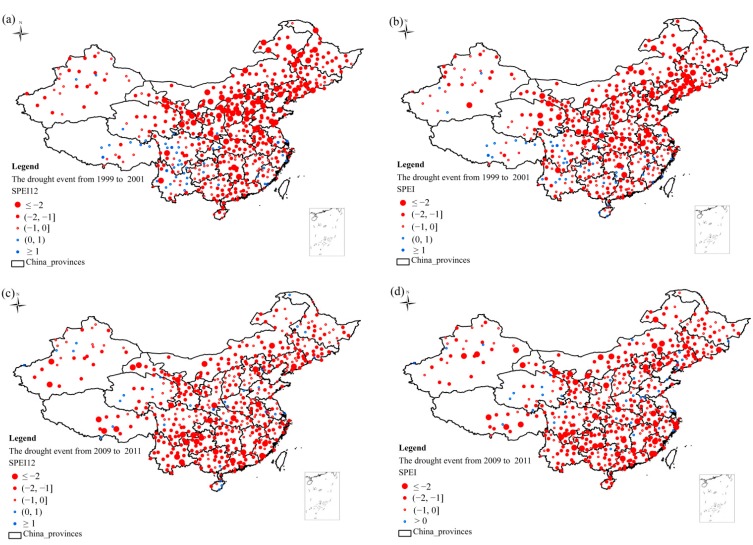
(**a**) The spatial distribution of large-scale drought events from 1999 to 2001 based on the SPEI-12; (**b**) The spatial distribution of large-scale drought events from 1999 to 2001 based on the SPEI_opt_; (**c**) The spatial distribution of large-scale drought events from 2009 to 2011 based on the SPEI-12; (**d**) The spatial distribution of large-scale drought events from 2009 to 2011 based on the SPEI_opt_.

Two large-scale drought events obtained from the temporal evolution of the SPEI time series. The first drought event happened during the period from 1999 to 2001, and the other occurred from 2009 to 2011 (Figure 2a and Figure 3a). The spatial distribution of these two large-scale drought events was shown in Figure 4. The severity of large-scale drought events from 1999 to 2001 based on the SPEI-12 (Figure 4a) was more serious than that employed the SPEI_opt_ for various vegetation types (Figure 4b).

The spatial pattern of drought with the SPEI-12 was mainly concentrated in Northeast China and also east of central China. The severity of the drought was more serious in Northeast China and lesser east of central China based on the SPEI_opt_. The drought that occurred from 2009 to 2011 based on the SPEI-12 (Figure 4c) had less clear pattern than the SPEI_opt_ (Figure 4d). The drought based on the SPEI_opt_ was more concentrated in Southern China and Northern China, especially in Northeast China. Central China, however, did not have obvious drought signals based on the SPEI_opt_.

**Figure 5:**
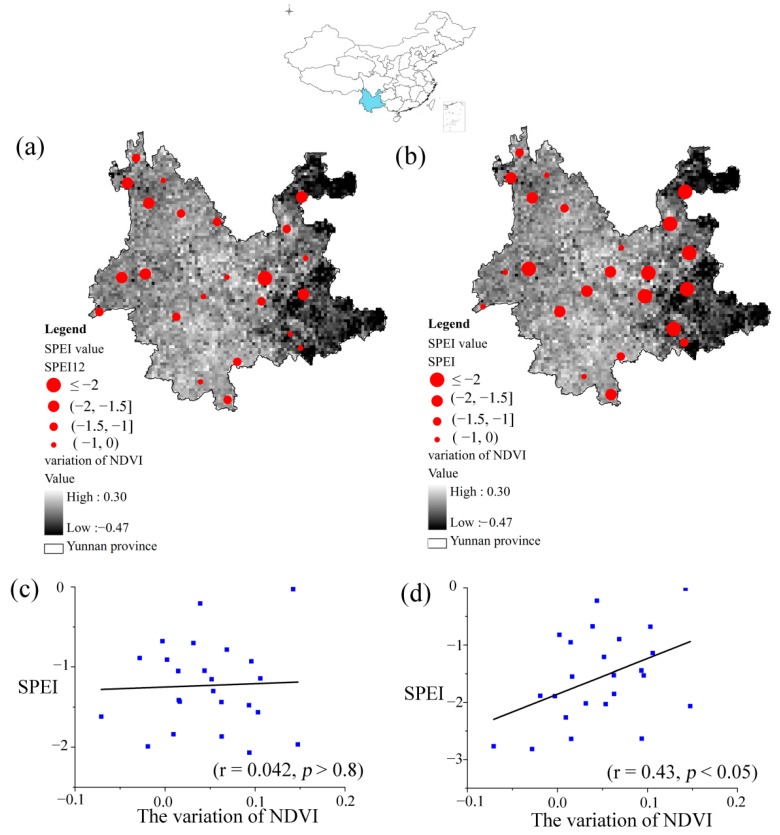
The extreme drought event in the Yunnan province based on the SPEI-12 (**a**) and on the SPEI_opt_ (**b**); The base map is the variation of NDVI in October of 2009; (**c**) The scatter plot based on the SPEI-12; (**d**) The scatter plot based on the SPEI_opt_. It considers both the growing season (April to October) and the time when the extreme drought condition happened.

An extreme drought event occurred in Yunnan Province from October of 2009 to March of 2010 due to decreased precipitation and increased temperature [55]. The SPEIs with both the SPEI-12 and the optimal time scale (SPEI_opt_) detected the extreme drought conditions (Figure 5a,b). Most meteorological stations based on the SPEI_opt_ detected the drought (SPEI ≤ −1), while the drought detected by the SPEI-12 did not show obvious drought conditions. The correlation between the NDVI_anomaly_ and the SPEI-12 was not significant (Figure 5c) which indicated that the SPEI-12 could not reflect the impact of drought on the variations of NDVI, while the NDVI_anomaly_ and the SPEI_opt_ showed a significant positive correlation (r = 0.425, *p* < 0.05) (Figure 5d), The drought based on the SPEI_opt_ also showed a clearly spatial difference from the west to the east, and the droughts in the middle and east were much more serious than that in the west. The spatial pattern and the severity of the drought based on the SPEI_opt_ were in line with previous studies concerning the drought in Yunnan [55,56].

### 3.4. Regression Models Between Vegetation Growth and Drought with Both SPEI-12 and SPEI_opt_

**Figure 6:**
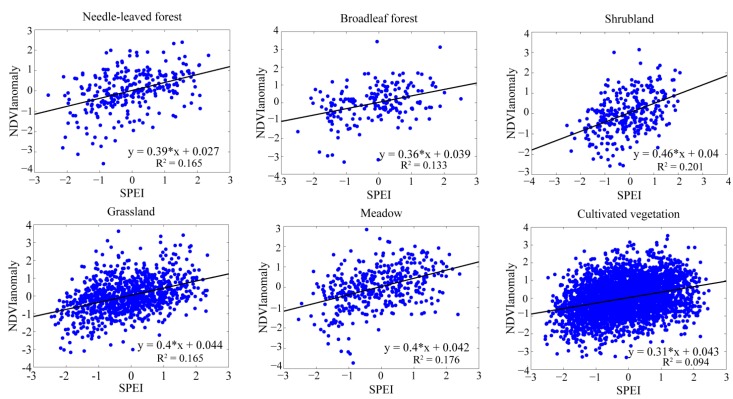
The responses of vegetation to the drought based on the SPEI-12.

**Figure 7:**
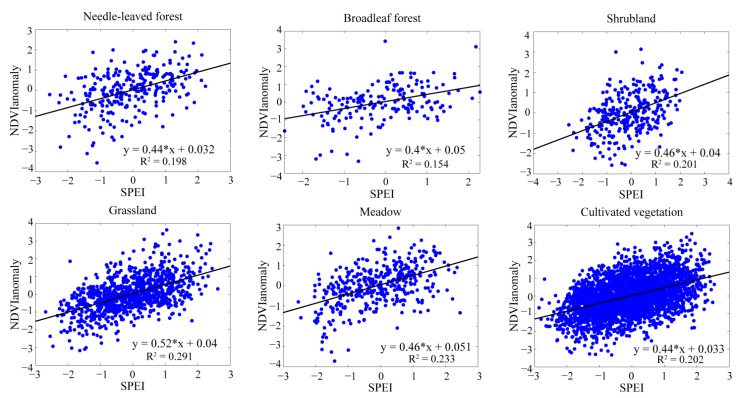
The responses of vegetation to the drought based on the SPEI_opt_.

To compare the responses of various vegetation types to the drought during the growing season, the regression models between the NDVI_anomaly_ and the time series of SPEI with both the optimal time scales and the SPEI-12 were employed. The regression analysis between the anomaly of NDVI and the SPEI_opt_ (SPEI-12) could reflect the responses of vegetation to drought, and all the vegetation types passed the significance test. Based on the SPEI-12 (Figure 6), the vegetation types located in both the wet regions and the arid regions showed similar results, and the differences among all the vegetation types were not evident. The responses of vegetation to the drought based on the SPEI_opt_ (Figure 7) showed better results than the SPEI-12. The coefficients of determination (R^2^) for all the vegetation types have increased (2.1% to 12.6%), except for shrubland. The vegetation types such as grassland, meadow and cultivated vegetation located in the arid sites had a higher percentage of increased R^2^ than the other three vegetation types. They were related to the short time scales and usually had water deficits during the growing season, so they were more sensitive to the water conditions. The forests were usually located in water surplus sites and corresponded to the long time scales close to the SPEI-12; thus, the R^2^ improvement was relatively small.

## 4. Discussion

The SPEI with various time scales is a new climatic drought indicator that could contain information on the evapotranspiration on drought, thereby making it possible to reflect the changes in the water demands under the background of global warming [23]. This study aimes to determine the optimal time scales of SPEI (SPEI_opt_) for various vegetation types, and further analyze the spatio-temporal characteristics of the drought and compare two large-scale drought events and extreme drought events based on the SPEI with both the SPEI-12 and the optimal time scales (SPEI_opt_). The covariation between the SPEIs with various time scales (1–12 months) and the anomalies of the normalized difference vegetation index (NDVI) is employed to determine the optimal time scales for various vegetation types. To the best of our knowledge, this is the first study to reflect the differences among various vegetation types based on the different SPEI time scales in China. The responses of vegetation to the drought are different for each land covers and the different responses of vegetation to the drought are investigated based on the regression models between the anomaly of NDVI and the SPEI with the optimal time scales.

Humid vegetation types, such as needle-leaved forest, broadleaf forest and shrubland, and arid vegetation types, such as grassland, meadow and cultivated vegetation, are all affected by drought, which indicate that the persistence of water deficit (*i.e.*, the drought time scale) could play a significant role in determining the sensitivity of vegetation types to drought. For needle-leaved forest, broadleaf forest and shrubland, the optimal time scales are related to long time scales from 10 to 12 months. This indicates that the forests could be insensitive to short droughts, but the long and intense drought could affect their physiological structure [57,58]. These vegetation types may have conservative water and deeper root systems that can mitigate the effects of short water shortages on vegetation [59,60].

The optimal time scales for grassland, meadow and cultivated vegetation correspond to short time scales from 2 to 4 months. The arid vegetation types have rapid reactions when water deficits below the normal conditions occur, and they could be explained by the fact that the vegetation types are more sensitive to atmospheric drought [61] and have physiological anatomical and functional strategies to rapidly adapt to the changing water availability [33,62].

The correlation coefficients based on the optimal time scales for various vegetation types are higher than that based on the fixed time-scale (SPEI-12). For the forests, such as needle-leaved forest and broadleaf forest, the increased proportions of the correlation coefficients are relatively lower. This could be explained by the fact that they all related to the time lags from 10 to 11 months, which is close to the SPEI-12. The forests exhibit strong responses to the drought with long time scales (10–12 months) [33], and the determination of the optimal time scales could also reflect the differences in the forest types. The grassland, the meadow and the cultivated vegetation have increased correlation coefficients, mainly in that they respond to the short time scales (2 to 4 months), which show evident differences compared with the SPEI-12. The shorter time scales show the rapid responses of these three vegetation types to the drought, and as the time scales increased to medium and long time scales, the responses are weaker [26]. These results are meaningful in that they show the significance of detecting the optimal time scales for various vegetation types.

The spatio-temporal characteristics of drought based on the SPEI with the optimal time scales for all the vegetation types show that the frequency of drought has increased during the 21st century, and a drying trend has occurred in most of China. This result is similar to those of previous studies employing various other methods to investigate the drought situation in China [15,16,63]. Two large-scale drought events based on the SPEI with both the SPEI-12 and the optimal time scales (SPEI_opt_) show discrepancies. The large-scale drought event from 1999 to 2001 is mainly concentrated in Northern China, where the major vegetation types are grassland and cultivated vegetation, with relatively short time scales; thus, there exist evident differences between the two SPEI time scales and the severity of drought is less serious based on the short time scales. The large-scale drought event from 2009 to 2011 is distributed in North China and South China. The situation in North China is more serious based on the SPEI-12, while Southern China does not show clear differences, mainly because forests and shrubland are located in these regions with related long time scales close to the SPEI-12. The extreme drought event in Yunnan Province shows a better spatial pattern based on the SPEI_opt_, and these results are in line with many studies concerning the extreme Yunnan Province drought event [55,56].

The regression models between the NDVI_anomaly_ and the SPEI with both the SPEI-12 and the optimal time scale (SPEI_opt_) for various vegetation types are compared, and the results show that the relationship between the NDVI_anomaly_ and the SPEI_opt_ has better linear trends, as the R^2^ increased by 2.1% to 12.6%. This further indicates that the vegetation type-specific SPEI_opt_ for various vegetation types is better at showing the differences in the responses of water deficits. The grassland, meadow and cultivated vegetation display increased R^2^ values relative to the other three vegetation types in that they are related to the short time scales (2–4 months) that have clear differences with the SPEI-12. The water deficits with short time scales have large impacts on vegetation located in the arid/sub-arid sites where the water balance constrains the main physiological processes of vegetation relative to mesic sites [19,27,64,65,66,67]. The forests have relatively smaller increases in R^2^ in that they are related to the long time scales close to the SPEI-12. The water conditions are no longer the limiting factor for vegetation growth, and temperature and radiation are much more important [21].

The results show that the impact of drought on vegetation varies among different vegetation types. The aridity and associated characteristics could also explain the spatial differences of the drought. There are still other factors such as wildfire disturbances, tree mortality and land cover change that could lead to NDVI decreases. Ruling out these infrequent incidents should prove helpful to study drought in further studies. Given the large spatial heterogeneity of China, additional studies are needed to assess the impact of local conditions, such as the topography, on the vegetation’s responses to the drought at different time scales. Our findings are helpful in ecological assessments and understanding the impacts of drought on vegetation.

## 5. Conclusions

The climatic drought index (SPEI) derived from meteorological observations was extensively used to evaluate potential impacts of water deficits on terrestrial ecosystems. In this study, we estimated the optimal time scales for different vegetation types and found that forests had much longer time scales (10 to 12 months) than those of grassland, meadow and cultivated vegetation (2 to 4 months), which implied that the prior precipitation had different impacts on different vegetation types. When the vegetation type-related optimal time scales were used, the SPEI could better reflect the vegetation’s responses to water conditions, with the correlation coefficients between SPEIs and NDVI anomalies increased by 5.88% to 28.4%. The SPEI with the optimal time scales further indicated that the extent of drought across the entirety of China displayed an increasing trend during the period from 1982 to 2011, with the frequency of drought increasing during the 21st century and the drying trend occurring in most of China. The regression analysis between the anomaly of NDVI and the SPEI with the optimal time scales for various vegetation types showed that R^2^ increased by 2.1% to 12.6%. These results are helpful in assessing the sensitivity of vegetation to the drought and adapting appropriate management strategies to mitigate the impact of increasing droughts on vegetation growth.
